# Low-Toxic, Earth-Abundant Nanostructured Materials for Thermoelectric Applications

**DOI:** 10.3390/nano11040895

**Published:** 2021-03-31

**Authors:** Farheen F. Jaldurgam, Zubair Ahmad, Farid Touati

**Affiliations:** 1 Department of Electrical Engineering, College of Engineering, Qatar University, Doha 2713, Qatar; fj1912900@student.qu.edu.qa (F.F.J.); touatif@qu.edu.qa (F.T.); 2 Qatar University Young Scientist Center (YSC), Qatar University, Doha 2713, Qatar; 3 Center for Advanced Materials (CAM), Qatar University, Doha 2713, Qatar

**Keywords:** thermoelectric materials, nanostructures, earth-abundant, low-toxic, figure of merit, Seebeck coefficient

## Abstract

This article presents recent research directions in the study of Earth-abundant, cost-effective, and low-toxic advanced nanostructured materials for thermoelectric generator (TEG) applications. This study’s critical aspect is to systematically evaluate the development of high-performance nanostructured thermoelectric (TE) materials from sustainable sources, which are expected to have a meaningful and enduring impact in developing a cost-effective TE system. We review both the performance and limitation aspects of these materials at multiple temperatures from experimental and theoretical viewpoints. Recent developments in these materials towards enhancing the dimensionless figure of merit, Seebeck coefficient, reduction of the thermal conductivity, and improvement of electrical conductivity have also been discussed in detail. Finally, the future direction and the prospects of these nanostructured materials have been proposed.

## 1. Introduction

An ideal thermoelectric (TE) material should have the thermal properties of the glass and electrical properties of a crystal, also known as “phonon glass electron crystal” [1]. A common strategy for improving the performance of the conventional TE materials is to reduce thermal conductivity by introducing nanometer-scale grains or interfaces that scatter the heat-carrying phonons [2,3,4]. But improving the electrical conductivity and decreasing the thermal conductivity of materials is always a challenge due to their interdependent nature. In the nanostructured materials, the lower lattice thermal conductivity is observed because of the efficient scattering of phonons over an extensive mean free path range by a high density of interfaces. The efficiency of a TE material can be defined in terms of the figure of merit(Z) of the materials [5] is given by:

(1) $$Z=\frac{{\left(\alpha_{p}-\alpha_{n}\right)}^{2}}{{\left({\left(k_{p}\cdot \rho_{p}\right)}^{1/2}+{\left(k_{n}\cdot \rho_{n}\right)}^{1/2}\right)}^{2}}$$

where $\alpha_{p}\&\alpha_{n}$ are the Seebeck coefficients of the two materials of the thermocouple, $\rho_{p}\&\rho_{n}$ are the electrical resistivities, and $k_{p}\&k_{n}$ are the thermal conductivities.

*ZT* is the product of the average temperature and the figure of merit and is called the dimensionless figure of merit and for a single material can be given by:

(2) $$ZT=\frac{\alpha^{2}T}{k\rho }=\frac{\alpha^{2}\sigma T}{k}$$

(3) $$T=\left(T_{H}-T_{C}\right)/2$$

where, $T_{H}$ is the hot side temperature, $T_{C}$ is the cold side temperature of the modules, σ and k are the electrical and thermal conductivity of the material, respectively.

The maximum ZT has been achieved by reducing the thermal conductivity through nanostructured engineering or hierarchal architecting [6]. Nevertheless, the high cost and toxicity are barriers to the real-life application of these nanostructured materials in TEGs [7]. The preliminary materials that were being used for TE applications were tellurides doped with bismuth, antimony, and lead, etc. However, due to their high cost and toxicity, these TE materials were only used for cooling and power generation applications, where the price was not the primary concern. Later, with the discovery of silicon-germanium (SiGe), the TEGs were principally employed to convert heat into power in spacecraft designed for deep-space NASA missions. Due to these materials’ expensiveness and toxicity, it has not been possible to utilize them in widespread and daily-life applications. Hence, it was highly desirable to develop low-cost and low-toxic TEGs with high ZT value, using materials that are abundant, low toxic, and easy to synthesize. Thus, TE materials such as clathrates, skutterudites, half-Heuslers, oxide, silicides, chalcogenides, and selenides have been proposed. Elemental doping, nanostructuring, nanowiring, nano-inclusion, and ball milling techniques have been used to enhance the ZT value in these materials.

This review spotlights materials made from sustainable, Earth-abundant, cost-effective, and low-toxic elements that have the potential to replace conventional lead telluride, i.e., skutterudites made from rare-earth elements. These TE materials can provide a prospective low-cost solution to deal with the energy crisis by utilizing waste heat and concentrated solar power. In these TE materials, the rattlers, vacancies, and voids can significantly reduce thermal conductivity and improve the ZT value. The “phonon glass electron crystal” characteristics of these cost-effective and non-toxic materials can remarkably be enhanced by producing their nanowires and nanocomposites. Here it is important to mention that in this review the choice of the materials was done based on the factor which include cost, performance, and relatively low toxicity. Of course, the toxicity of the material is sometimes difficult to completely avoid if we consider all three factors.

## 2. Recent Advancements

**Figure 1:**
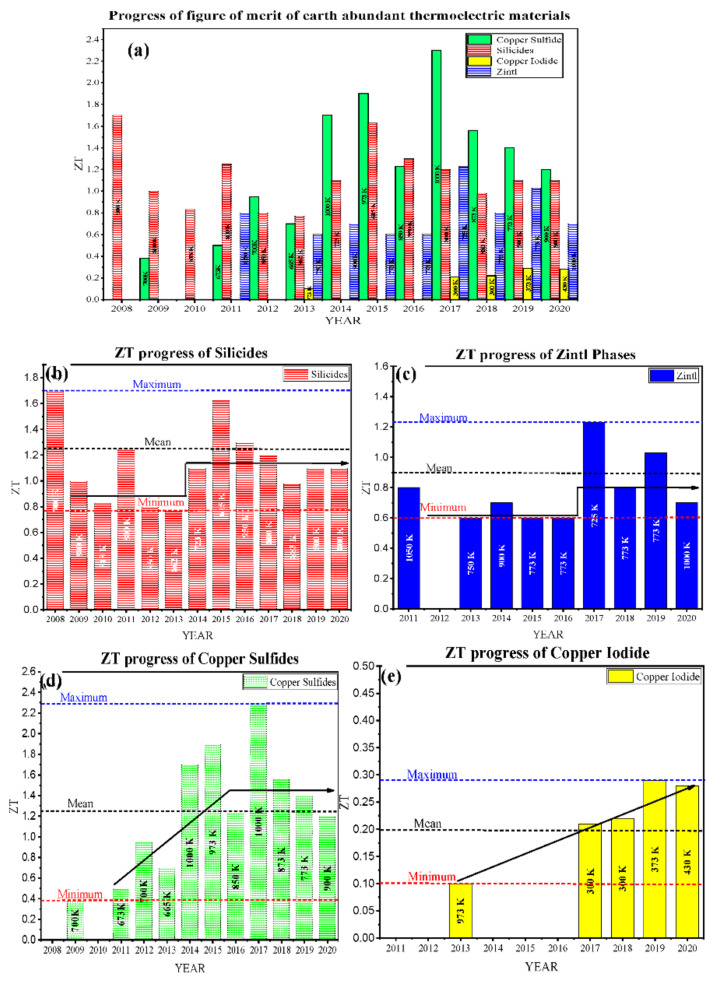
(**a**) Recent progress of figure of merit (ZT_max_) of Earth-abundant low-toxic thermoelectric materials; Low-temperature thermoelectric materials (copper iodide and its alloys), Intermediate and high-temperature thermoelectric materials (copper sulfides, silicides, Zintl compounds, and their alloys) from the year 2008–2020. The temperatures at which the maximum ZT recorded is mentioned in the bar-charts. (**b**) silicides, (**c**) Zintl compounds, (**d**) copper sulfides, (**e**) copper iodide (Tables 1 and 2).

Figure 1a represents recent advancements in the low-toxic and earth-abundant TE materials, and their figure of merit (ZTmax) attained at low, intermediate, and high temperatures. These materials are sufficiently available and harmlessly disposable and consist of various low toxic silicides, Zintl compounds, and copper-base alloys, including copper sulfides and copper iodides. These materials possess enormous advantages and have room for further improvements, making them potential competitors to the traditional TE materials. For instance, silicides such as silicon germanium are available since the first synthesis of thermoelectric materials. They are mainly used in space applications, with the only drawback of germanium’s high fabrication cost, but over time, the researchers have resolved this issue by using lower amounts of germanium or replacing germanium with low-toxic and abundantly available materials such as magnesium, manganese, chromium, etc. Along with silicon, these materials form various silicides that have both the benefits of traditional silicides and eco-friendliness. The ZT value for such silicides has been successfully attained >1; even, in some cases, it has been reported to be over 1.5 (see Figure 1b).

Similarly, the Zintl compounds are a subgroup of intermetallic compounds, initially comprising toxic and rare-earth materials, but lately, a few Zintl compounds, such as Ca_3_AlSb_3_, Mg_3_Sb_2_, CaAl_2_Si_2_, etc., have also been developed that have the benefits of Zintls as well as low toxicity and abundancy. Low-toxic Zintls likewise have good potential for commercialization for heat recovery applications. The average ZT value for such materials lies in the range of 0.8 ± 0.2, as shown in Figure 1c. Figure 1d shows that copper sulfides (with a mean ZT value >1.2) also exhibit great potential for the intermediate and high-temperature range of low-toxic and geo abundant thermoelectric materials. In a case study of copper sulfides doped with trace amounts of rare-earth chalcogenides, the highest ZT value >2 is reported. However, low values of ZT have been observed in copper iodide and its alloys, as shown in Figure 1e. In the low-temperature range applications, copper iodide is a transparent and flexible thermoelectric material. Its maximum ZT value is deficient (~0.3) compared to other TE materials in this temperature range.

## 3. Temperature-Dependent Classification of Low-Toxic Earth-Abundant Thermoelectric Materials

The TE materials are classified into three categories based on their operating temperatures: low temperature, medium or intermediate temperature, and high temperature. In this section, we discuss the temperature-dependent types of low-toxic and earth-abundant thermoelectric materials.

### 3.1. Low-Temperature Thermoelectric Materials

**Figure 2:**
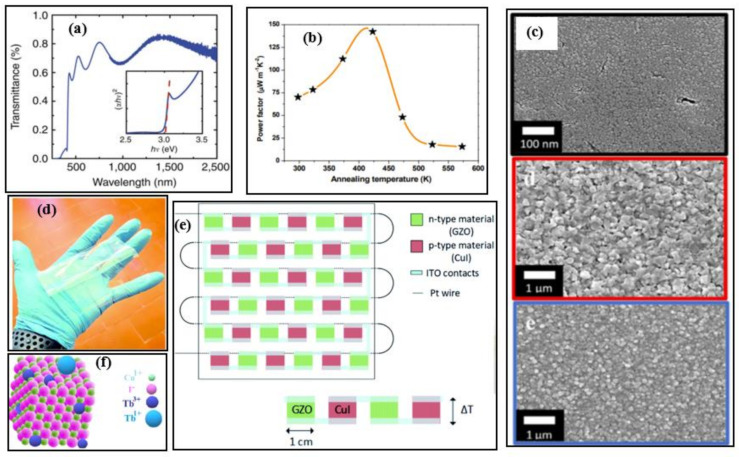
(**a**) Transmittance spectra of copper iodide thin film deposited of glass (thickness of 300 nm), Reproduced with permission from [9]. Copyright © 2017, Nature Communications, (**b**) Room temperature power factors of copper iodide at different annealing temperatures, Reproduced with permission from [10]. Copyright © 2018, Wiley Online Library, (**c**) SEM image of nanostructured CuI synthesized using vapour iodination and thermal evaporation, Reproduced with permission from [11]. Copyright © 2018, Scientific Reports Nature. (**d**) A 10 × 10 cm^2^ Kapton-based copper iodide (CuI) and gallium-doped zinc oxide (GZO) based thermoelectric generator prototype, Reproduced with permission from [12]. Copyright © 2019, Royal Society of Chemistry, (**e**) Schematic representation of transparent flexible copper iodide thin-film thermoelectric generator (p-type material is CuI and n-type material is GZO) with 17 p-n modules connected in series with indium tin oxide (ITO) using platinum wire, Reproduced with permission from [12]. Copyright © 2019, Royal Society of Chemistry, (**f**) Illustration of CuI (doped with terbium) single nanocrystal, Reproduced with permission from [13]. Copyright © 2020, Elsevier.

In a low-temperature range, copper iodide (CuI) is a promising candidate as a low-toxic and Earth-abundant material. CuI can be a potential alternative to the commercially available low-temperature TE materials such as bismuth telluride. It is abundantly available and can find its applications in smart screens or windows, portable and wearable energy devices due to its flexible and transparent nature [6,8,9]. It can be seen from Figure 2a the optical transmittance spectra of the CuI exhibits ~60–85% transmittance in the wavelength range 410–2000 nm. High power density with good mechanical flexibility has been noticed in the prototype CuI-based module [10]. The Seebeck coefficient and power factor were attained up to ~431 µVK^−1^ and ~70 µWm^−1^K^−2,^ respectively, by tuning the defect iodine chemistry via thermal annealing; the best thermoelectric properties were observed at moderate annealing temperatures (~430 K) [10] as illustrated in Figure 2b.

Typically, the transparent TE materials show low electrical conductivities resulting in inferior TE properties. However, a transparent TEG consisting of p-type CuI and n-type gallium doped zinc oxide (GZO) with high electrical conductivity 110 Scm^−1^ and ZT_max_ of 0.22 at 300 K, has been fabricated by Faustino et al. [11]. The GZO thin film was prepared using different fabrication including, solid iodization, thermal evaporation, and vapor deposition method. The SEM images of nanostructured CuI synthesized using the above-mentioned methods are depicted in Figure 2c. A clear difference in the size of the nanostructures is observed, and this study reveals that the CuI film prepared by solid iodination of Cu works better in both TE characteristics and in terms of optical transparency. Later on, Coroa et al. [12] constructed a transparent flexible thermoelectric generator using 17 p-n modules on a polyimide Kapton^®^ CS substrate, where CuI and GZO were used as p-type and n-type materials, respectively as shown in Figure 2d and sketched in Figure 2e.

Further, 300 nm thick CuI film doped with terbium (Tb) single nanocrystal demonstrated a ZT_max_ value of 0.29. The illustration of the Tb-doped CuI structure is given in Figure 2f, which can be operated at 430 K [13]. The current ZT progress of CuI-based TE materials is given in Table 1. The maximum ZT of these materials is ~0.3, which is way less than other materials in this temperature range.

### 3.2. Intermediate Temperature Thermoelectric Materials

In the medium temperature range, TE materials (ranging between 500–900 K) include chalcogenides skutterudites, tin, half-heusler, etc. Chalcogenides such as sulfur alloys are appropriate low-toxic and low-cost materials in this temperature range and can be used for waste heat recovery. The sulfide-based chalcogenides investigated for the TE applications include copper sulfide (Cu_x_S_y_), copper zinc tin sulfide (CZTS), bismuth sulfide iodide (BiSI), silicon sulfide (SiS), and silicon selenide (SiSe).

#### 3.2.1. Chalcogenides (Sulfur-Based)

**Figure 3:**
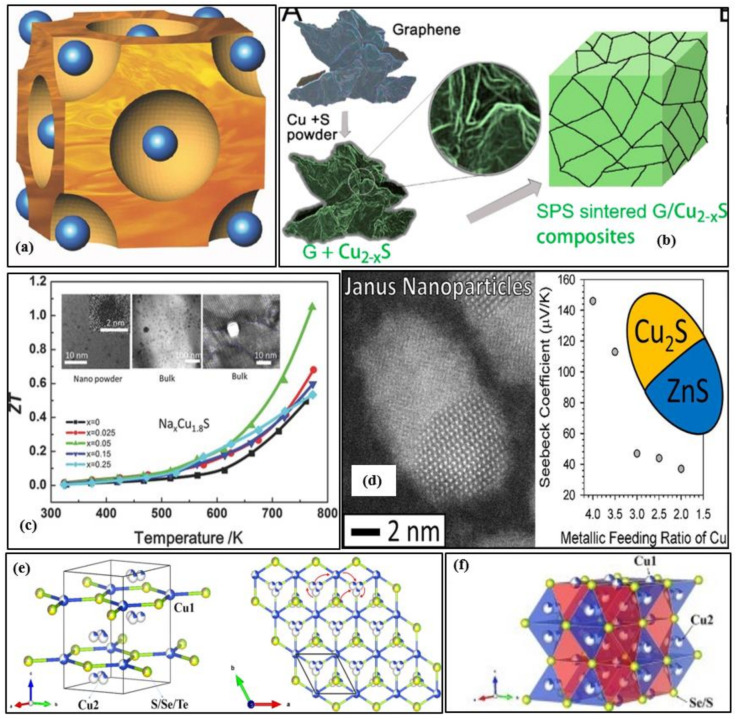
(**a**) The crystal structure of the cubic-chalcocite α phase of Cu_2−x_S (the sulfur atoms are represented by the blue spheres), Reproduced with permission from [39]. Copyright © 2014, Wiley Online Library, (**b**) Schematic representation of the spark plasma sintering process for fabricating graphene/Cu_2−x_S nanocomposites, Reproduced with permission from [22]. Copyright © 2018, Elsevier, (**c**) The temperature dependence of figure of merit ZT value for Cu_9_S_5_ sample with different sodium contents. The inset shows nanostructure in the TEM image of Na_1−x_Cu_9_S_5_ powders, Reproduced with permission from [31]. Copyright © 2016, Wiley Online Library, (**d**) Schematic Illustration of STEM-HAADF image of copper sulfide-zinc sulfide Janus nanoparticles at 2nm size and dependence of the Seebeck coefficient on the metallic feeding ratio of copper, Reproduced with permission from [35]. Copyright © 2016, ACS Publications. (**e**) Visualization of the crystal structure with a space group (P6_3_/*mmc*) at 100 K of Cu_2−y_S_1/3_Se_1/3_Te_1/3_ (Hexagonal structure), Reproduced with permission from [32]. Copyright © 2017, Elsevier., (**f**) Visualization of the crystal structure with a space group (P6_3_/*mmc*) at 100 K of Cu_2_Se_0.5_S_0.5_, Reproduced with permission from [33]. Copyright © 2017, Elsevier.

The properties and structures of copper sulfide-based thermoelectric materials such as chalcopyrite (CuFeS_2_), chalcocite (Cu_2_S), bornite (Cu_5_FeS_4_), kesterite (Cu_2_SnS_4_), stannite (Cu_2_FeSnS_4_), tetrahedrite [(Cu, Fe)_12_Sb_4_S_13_], and colusite [Cu_26_V_2_(As,Sn, Sb)_6_S_32_] have been reviewed [14]. Wei et al. presented a complete review of the family of copper chalcogenides (Cu_2_X, where X = S, Te, Se) from their structural features to design strategies to device development [15]. The primary concern is the volatilization of sulfur during synthesis and under higher temperature operations, thereby affecting the material stability. The loss of sulfur leads to changes in the nominal stoichiometries making reproducibility challenging to achieve. Also, the mobile copper ions migrate and form a layer at one end of the thermoelement under the electric field created when the device is operated at a temperature gradient resulting in lower performance, cracking, and compositional changes. A theoretical evaluation of the potential properties and stability of Fe-doped Cu_2_S was performed [16]. Some other works also showed this problem of Cu ions migration under working conditions [17,18,19,20]. One possible method to avoid this is by doping of bulk Cu_2_S with low mobility ions that can concurrently behave as electron-donors e.g., Fe [21]. Figure 3a shows the crystal structure of the chalcocite α phase of copper sulfide (Cu_2−x_S). The free copper ions travel and migrate freely in the sulfide sublattice (the blue spheres represent the sulfur atoms). Even though, in some case of Cu_x_S_y_ based TE materials, the ZT value has been exceeded over unity as shown in Figure 3c, however, mostly ZT > 0.6 is realized in p-type copper sulfides, but for n-type, the ZT value is even inferior. Tang et al. [22] suggested the introduction of a 3D graphene heterointerface via a combination of mechanical alloying and spark plasma sintering into the Cu_2−x_S matrix to improve the Seebeck coefficient and limit the thermal conductivity. A very high power factor and peak ZT of 1197 µWm^−1^K^−2^ and 1.56 at 873 K is attained for 0.75 wt%G/Cu_2−x_S with excellent tested reproducibility after five cycles [22]. Figure 3b shows the schematic illustration of the synthesis of G/Cu_2−x_S composite via spark plasma sintering. The addition of grain boundaries and interfaces in the Cu_2_S material through the dispersion of carbon nanotube(CNT) has restricted the thermal conductivity to less than 0.4 Wm^−1^K^−1^ at 448–798 K [23]. The Seebeck Coefficient reached up to 388 µVK^−1^ at 800 K due to the energy barrier filtering, and a peak ZT of 0.74 at 750 K was attained in the composite with a 10% CNT molar ratio [23]. Table 2 presents some prominent works in nanostructured copper sulfide materials for the past five years. Several novel methods were experimented to develop highly efficient nanostructured copper sulfides. Tang et al. [24] fabricated hexagonal sheet-like copper sulfide (Cu_2_S) nanocrystals of thickness 5–20 nm by the wet chemistry method and later densified by spark plasma sintering to obtain a high figure of merit of 0.62 at 773. This method can be used to synthesize large-scale copper sulfide bulk materials, and the results indicated that the phase structures and morphologies were highly influenced by the reaction time and temperature. Mulla et al. [25] suggested a novel rapid, and simple ambient chemical route to grow highly oriented and dense dendrites of Cu_2_S on a copper substrate and doping with copper ions to form uniform films covered with dense nanosheets. This cost-effective synthesis technique increased the Seebeck coefficient from ~100 to 415 µVK^−1^ and a high-power factor of ~400 µWm^−1^K^−2^.

Various materials such as sodium, tungsten, etc., are less toxic and widely available and are used as dopants to the nanostructured copper sulfides to enrich their thermoelectric properties. Single doping or dual doping leads to the introduction of nanoprecipitation or point defects or faults/stacking in the host structures, thereby influencing the thermoelectric performance. Cu_1_._8_S + 3 wt% In_2_S_3_ bulk samples were fabricated by doping with indium sulfide(In_2_S_3_) by spark plasma sintering to reach a peak ZT of 1.4 at 773 K [26]. The fast doping resulted in point defects, nanopores, and inclusion of second phases in the Cu_1_._8_S bulk materials [26]. These nanostructures dropped the thermal conductivity, and the indium doping enhanced the effective mass of the charge carriers improving the Seebeck coefficient [26]. Nanoscale CuInS_2_ phase was formed with the introduction of 2% indium sulfide (In_2_S_3_) to reach a ZT value of 1.23 and a relatively high-power factor of 1361 µWm^−1^K^−2^ at 850 K [27]. The introduction of Bi_2_S_3_ and Bi_2_S_3_@Bi core-shell nanostructured rods to the low-toxic geo abundant digenite (Cu_1_._8_S) has increased the maximum figure of merit to 0.77 at 723 K (in the case of 3% wt Bi_2_S_3_@Bi core-shell nanorods), which is significantly higher than the pristine Cu_1_._8_S [28]. Through the dispersion of 1.0 wt% SiC into the polycrystalline p-type Cu_1.8_S, a peak figure of merit of 0.87 at 773 K was attained by optimizing the carrier concentration, thereby reducing the thermal conductivity [29]. Doping the p-type Cu_1.8_S with tungsten diselenide (WSe_2_) has significantly enhanced the thermoelectric properties when compared to pristine p-type Cu_1.8_S (0.49 at 773) [30]. The WSe_2_ nanoparticles and their refined grain size have significantly decreased the thermal conductivity, and the optimized carrier concentration simultaneously enhanced the Seebeck Coefficient [30]. The peak ZT, Seebeck coefficient, and thermal conductivity of Cu_1.8_S + 1 wt% WSe_2_ sample at 773 K is 1.22, 110 µVK^−1,^ and 0.68 Wm^−1^K^−1,^ respectively [30]. The Na_x_Cu_9_S_5_(x = 0.025,0.05,0.15,0.25) nanopowders were sintered to fabricate p-type Na_x_Cu_9_S_5_ bulk materials via spark plasma sintering with an average size of 3 nm, and remarkable high ZT of ~1.1 and low thermal conductivity of 0.7 Wm^−1^K^−1^ at 773 K was observed by optimized carrier concentration and introduction of nanopores due to Na-doping (x = 0.05) [31]. Figure 3c shows the temperature dependence of the figure of merit ZT value for Cu_9_S_5_ samples with different sodium contents. The TEM image shown in the inset shows the nanostructure of Na_1−x_Cu_9_S_5_ powders.

**Figure 4:**
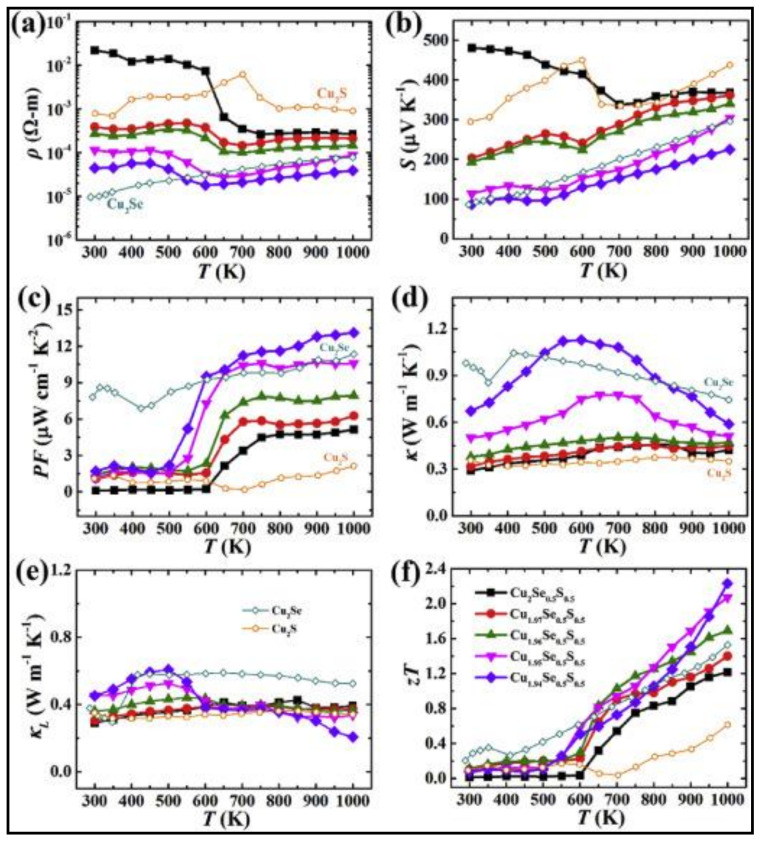
Temperature-dependent thermoelectric properties of Cu_2−y_Se_0.5_S_0.5_. (**a**) Electrical resistivity, (**b**) Seebeck coefficient, (**c**) power factor, (**d**) total thermal conductivity, (**e**) lattice thermal conductivity, (**f**) figure of merit ZT, Reproduced with permission from [33] Copyright © 2017, Elsevier.

Cu_2−y_S has exceptionally low thermal conductivity, while Cu_2−y_Se has efficient electrical transport properties. Combining these extraordinary characters into one solid material will result in exceptional thermoelectric performance. A nanoscale mosaic hexagonal structured component Cu_2−y_S_1/3_Se_1/3_Te_1/3_ solid solutions consisting of 10–30 nm-sized mosaic grains using three matrix compounds Cu_2_S, Cu_2_Se, and Cu_2_Te is synthesized to reach a superior ZT of 1.9 at 1000 K and y = 0.02 [32]. The schematic illustration of the hexagonal crystal structure of Cu_2−y_S_1/3_Se_1/3_Te_1/3_ is shown in Figure 3e. Each unit cell is shared with one S/Se/Te site and two copper sites. The projected plane representation of the crystal structure through the [001] direction can also be seen. The copper ions can travel among different copper sites, as indicated by the red arrow. The partial coloring of the atoms shows the atomic site occupancy. The reason for this exceptionally high ZT is its low thermal conductivity of ~0.49 Wm^−1^K^−1^ due to the phonon scattering by point defects, lattice strains of mosaic nanograins, and liquid-like copper ions. Zhao et al. [33] focused on this concept and formed a solid solution of Cu_2−y_Se_0.5_S_0.5_ with half sulfur and half selenium, and the resulting material possessed a unique hierarchical microstructure comprising of nanoscale domains, mesoscale polymorphs, and modulations. Figure 3f shows the visualization of the crystal structure of Cu_2−y_Se_0.5_S_0.5_ fabricated at 100 K. The disordered Cu2 sites are illustrated by blue tetrahedron and Cu1 sites by red octahedron. The tuning of its native Copper vacancies significantly improved its electrical transport properties and remarkably extraordinary thermoelectric performance with the maximum figure of merit of 2.3 at 1000 K and very low thermal conductivity of ~0.32 Wm^−1^K^−1^ at 1000 K is noted. This is up to date the highest value the figure of merit has reached in the case of chalcogenide bulk materials. Figure 4a–f summarize the thermoelectric properties of Cu_2−y_Se_0.5_S_0.5,_ namely the electrical conductivity, Seebeck coefficient, power factor, total thermal conductivity, lattice thermal conductivity, and figure of merit ZT. It is to be noted that Cu_2_Se_0.5_S_0.5_ had better electrical conductivity and Seebeck coefficient but lags in terms of power factor, and Z.T. Cu_1.94_Se_0.5_S_0.5_ had the best ZT value due to lower lattice conductivities at 1000 K. In another solid solution material, Cu_2_S_0.5_Te_0.5_, multiform nanostructures such as nano-domains of ordered structure, 4H polytype, mosaic nanocrystals, high density of stacking faults, and nanoscale periodic antiphase boundaries have been observed, resulting in ultra-low lattice thermal conductivity <0.4 and peak ZT of about 1.9 [34]. The multiformity of the material coincides with the phonon-glass-electron-crystal concept for extremely high-performance thermoelectric material.

Figure 3d shows the schematic illustration of the STEM-HAADF image of copper sulfide-zinc sulfide Janus nanoparticles (2 nm in size) and the dependence of the Seebeck coefficient on the metallic feeding ratio of copper. The Seebeck coefficient is high in the case of pristine CuS, and as the ZnS nanoparticle contents increase, the Seebeck coefficient decreases due to dilution of the material [35]. Copper zinc tin sulfide (CZTS) elements are low-toxic and extremely abundant. The electrical conductivity and figure of merit of CZTS have significantly improved, and thermal conductivity is lowered by doping the CZTS nanocrystal material with copper; however, there has been a decline in the Seebeck coefficient, and this may be due to the higher carrier concentration from copper doping. The copper tin sulfide (CTS) has the benefit of disordered metal atoms arrangement in the cubic and tetragonal phases, and it also has a distinct electronic nature that is influenced by the S-3p and Cu-3d orbitals, which gives place to independent variation of carrier concentration through heavy doping with no effect on the valence band. Based on these beneficial properties, a p-type CuSnS_3_ doped with zinc (20%) was synthesized using spark plasma sintering to form a 3-dimensionally conductive network for holes resulting in ultra-low thermal conductivity of ~0.9 ± 0.4 Wm^−1^K^−1^, and a high power factor of ~0.84 mWm^−1^K^−2^ at 723 K [36]. The addition of tin in the pristine Cu_1.8_S introduces a second phase to optimize the carrier concentration and formation of nanopores to reduce the thermal conductivity k. An ultra-low thermal conductivity and a high ZT value of 0.30 W/mK and 0.81 respectively at 773 K is recorded in the case of Sn_0.01_Cu_1.79_S bulk material [37]. Two heterogeneous phases (Cu_12_Sb_4_S_13_ and Cu_4_SnS_4_) were induced due to Sb/Sn co-doping that rapidly declined the carrier concentration and enriched the phonon scattering to record a high ZT of 1.2 at 773 K for Cu_1.8_Sb_0.02_Sn_0.03_S bulk sample [38].

**Table 2:** Prominent copper-sulfide nanostructured alloys/composites reported from 2016–2020.

Sr.no	Material	Operating Temperature (K)	Synthesis Technique	σ * (Scm^−1^)	k * (Wm^−1^K^−1^)	Seebeck Coefficient (µVK^−1^)	PF * (S^2^σ/µWm^−1^K^−2^)	ZT *_max_	Year/Reference
1	20%Zn-Doped Cu_2_SnS_3_	723	Spark plasma Sintering	~600	0.9 ± 0.4	~100	840	0.58	2016 [36]
2	Na_0.05_Cu_9_S_5_	773	Mechanical Alloying Spark Plasma Sintering	~850	0.7	~110	1050	1.1	2016 [31]
3	Cu_2_S with 2%wt In_2_S_3_	850	Spark Plasma Sintering	~500	0.95	200	1361	1.23	2016 [27]
4	Sn_x_Cu_1.8-x_S at x = 0.01	773	Planetary ball mining Spark Plasma Sintering	19	0.4	250	~500	0.81	2017 [37]
5	Cu_1.98_S_1/3_Se_1/3_Te_1/3_	1000	Spark Plasma Sintering	N/A	~0.49	250	1120	1.9	2017 [32]
6	Cu_2_S NS doped with Cu ion	300–360	Simple room temperature chemical route	N/A	N/A	415	~400	N/A	2017 [25]
7	Cu_2−y_Se_0.5_S_0.5_	1000	Spark Plasma Sintering	N/A	0.32	370	1500	2.3	2017 [33]
8	αCu_2_S (1–7 µm) βCu_2_S (5–20 nm)	573 773	Hydrothermal synthesis Wet chemistry method	~100 ~1000	~0.2 ~1.23	~420 101	196 985	0.38 0.62	2017 [24]
9	Cu_2.14_Co_0.8_Mn_0.05_SnS_4_ (Cu/Mn co-doping)	800	Mechanical Alloying Hot Pressed Sintering	N/A	~1.0	~200	1026	0.8	2017 [40]
10	Cu_2_S_0.5_Te_0.5_	-	Spark Plasma Sintering	N/A	<0.4	N/A	N/A	1.9	2017 [34]
11	Cu_1.8_S with 3% wt Bi_2_S_3_@Bi core-shell nanorods	723	Mechanical Alloying Spark Plasma Sintering	~1500	0.91	85	~990	0.77	2017 [28]
12	Polycrystalline p-type Cu_1.8_S with 1.0 wt% SiC NP *	773	Mechanical Alloying Spark Plasma Sintering	~900	0.7	115	~900	0.87	2017 [29]
13	Cu_1.0_Fe_0.9_7S_2.12_(S_1.96_)(Fe−1-S) n-type	623	Hot injection Sintering	850	0.5	238	N/A	0.13	2017 [41]
14	Cu_5_FeS_4_ Bornite NP	320	Mechanical Alloying Annealing	N/A	0.46	130	250	0.28	2018 [42]
15	Cu_2−x_S with 0.75%wt graphene	873	Mechanical Alloying Spark Plasma Sintering	~500	0.67	~165	1197	1.56	2018 [22]
16	Cu_2_Sn_1−x_Zn_x_S_3_ NP *	670	Chemical Synthesis Pulse electric Current Sintering Method	95.2	0.45	218.2	~450	0.64	2018 [43]
17	Cu_1.8_S+ 1wt%WSe_2_ NP *	773	Mechanical Alloying Spark Plasma Sintering	~800	0.68	110	~900	1.22	2018 [30]
18	Cu_2_S with carbon nanotube	700–800	One-step ultrasonic reaction method	~50	<0.4	388	~500	0.74	2019 [23]
19	3%wt In_2_S_3_ doped Cu_1.8_S	773	Spark Plasma Sintering	~600	0.65	110	~1100	~1.4	2019 [26]
20	Cu_1.8_Sb_0.02_Sn_0.03_S	773	Mechanical Alloying Spark Plasma Sintering	N/A	~0.6	175	975	~1.2	2019 [38]
21	Cu_1.8_S + 2 wt% Na_2_S	773	Mechanical Alloying Spark Plasma Sintering	~900	~0.9	106	~1048	0.78	2020 [44]
22	Cu_26_V_2_Sn_6_S_32_ NP *	673	Mechanical Alloying Plasma sintering	N/A	0.27	87	900	~0.7	2020 [45]
23	Ni_x_ Doped Cu_1.9_S at x = 0.02	773	Planetary Ball mining Spark Plasma Sintering	~500	1.08	149	1310	0.9	2020 [46]
24	Cu_2_SnS_3_ at 500 thermal temp	700	High energy reactive ball mining	N/A	0.26	350	110	0.30	2020 [47]
25	Cu_1.8_S_0.875_Te_0.125_	623	Mechanical Alloying Room temperature high-pressure sintering	~1000	0.82	90	630	0.352	2020 [48]

* Nomenclature: NP: nanoparticles; NS: nanosheets; σ: electrical conductivity; k: thermal conductivity; PF: power factor; ZT: the figure of merit.

**Figure 5:**
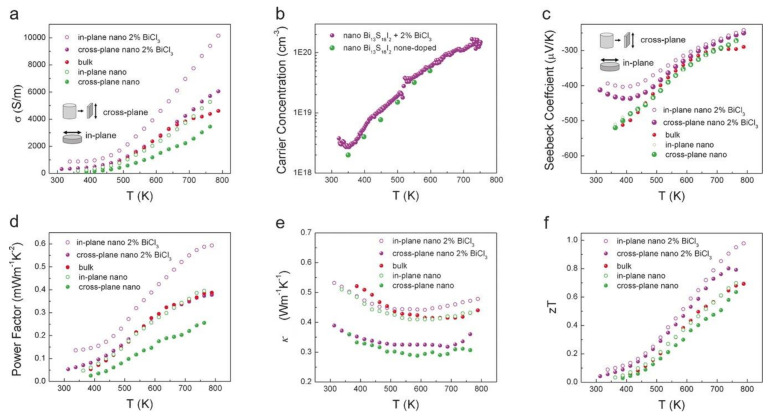
Temperature-dependent thermoelectric performance of the three Bi_13_S_18_I_2_ samples (bulk, nano & nano with 2% BiCl_3_) (**a**) Electrical conductivity, (**b**) Hall Carrier concentration, (**c**) Seebeck Coefficient, (**d**) Power Factor, (**e**) Thermal Conductivity, (**f**) Figure of merit (ZT), Reproduced with permission [49]. Copyright © 2018, Wiley Online Library.

Sulfide-based thermoelectric materials are studied mainly because of their low-cost and eco-friendly nature. However, the high thermal conductivity attributed to the lower atomic weight and low carrier mobility has led to inferior performance. Xu et al. [49] constructed a novel Bi_13_S_18_I_2_ using a solution synthesis resulting in the enhancement of the figure of merit for n-type material to 1 at 788 K (ZT = 1.0). Figure 5 shows the thermoelectric properties- electrical conductivities, carrier concentration, Seebeck coefficient, power factor, total thermal conductivities, and the figure of merit of bulk and nano BiCl_3_ samples. The study of TE properties of these material show that the electrical conductivities have improved with ascending temperature, 313–788 K and negative Seebeck coefficient values were observed, implying electron to be the majority carrier. The BiCl_3_ doped nano Bi_13_S_18_I_2_ sample displayed a better power factor compared to the undoped samples, and the thermal conductivities significantly decreased with temperature in almost all the samples [49]. The major drawback, in this case, was higher carrier concentration resulting in a low Seebeck coefficient. A higher power factor can be attained by fine-tuning the appropriate doping.

SiS and SiSe are two encouraging low-toxicity and geo-abundant candidates for intermediate temperature range thermoelectric applications. These materials have intrinsically low thermal conductivities because of their low frequency overlapped phonon modes and high-power factors due to their sharp peaks of state densities near the Fermi energy. A maximum ZT value of 1.99 and 1.06 has been noticed for SiSe, and SiS monolayers at T = 700 K and T = 500 K, respectively, and their thermoelectric properties were calculated using the first-principles calculation method in their ground pmma structure [50].

The peak ZT values are achieved at the electron chemical potential positions of about 0.05 eV and 0.10 eV above the valance band maximum or below the conduction band maximum of the materials. Tin selenide (SnSe) is a simple compound with ultra-low conductivity, gaining extensive interest for research among the thermoelectric community, and a ZT value as high as >2 has been reported [51]. Even though tin selenide is low-toxic, it is composed of rare-earth chalcogenide selenium, making it not so cost-effective. An alternate and advanced material SnS crystal with maximum ZT > 1.0 at 873 K and power conversion efficiency of ~10.4% has been suggested [52]. Doping SnS with 2% sodium(Na_0.02_Sn_0.98_S) has given ZT~1.1 at 870 K [53]. A high-performance sample of composition SnS_0.91_Se_0.09_ of ZT~1.6 at 873 K and power conversion efficiency ~18% were constructed with the introduction of selenium in tin sulfide crystals [54]. These works have paved the way for further research and study in layered materials for high performance in medium to high-temperature thermoelectric applications.

#### 3.2.2. Skutterudites

Skutterudites are a kind of cobalt arsenide minerals consisting of variable traces of iron or nickel substituting for cobalt to formulate CoAs_3_. These are lead and tellurium-free thermoelectric materials that are highly efficient for intermediate temperature range applications. Several skutterudites TE materials are compositions of low-toxic and earth-abundant materials (e.g., Co_4_Sb_12_, CeFe_4_Sb_12_). Rull-Barvo et al. [55] conducted an extensive study on skutterudites in terms of their efficiencies, scalability, stabilities, and applications. These materials have the figure of merits values close to 1.3 for p-type and 1.8 for n-type with good thermal and mechanical stabilities [56]. Liu et al. [57] reviewed the recent progress made in CoSb_3_-based materials and synergistic optimization of the thermal and electrical properties. Multi-filled skutterudites demonstrated inferior thermal conductivities resulting in a considerable increase of ZT values [58]. Reproducibility of high ZT values with stability has been challenging for skutterudites TE materials. Future works require ZT greater than 2 and a fine balance between high electrical properties with low thermal characteristics. These developments would undeniably make skutterudite a leading low-toxic and Earth-abundant TE material for the intermediate temperature application.

#### 3.2.3. Half-Heusler

Half-Heusler (HH) compounds are usually magnetic intermetallics with exciting thermoelectric properties with a composition XYZ, where Z is the element from the main group and X and Y are transition elements. RNiSn-type HH compounds are the most studied materials for TE applications, where R indicates the refractory metals Zr, Hf, and Ti. These materials (except Hr-based HH) are low-toxic and are available in large quantities. Casper et al. [59] reviewed the structural characterization, band gap origin, and operations of the semiconductor HH compounds for energy generation applications and spintronics. Nanocomposite approach, ternary HHs, and enhanced alloy scattering are some of the methods employed to improve the efficiency of the HH materials [60]. Hf-free low-toxic TiNiSn-based HH alloys were prepared with ZT in the range 0.9–1.2 [61,62]. Poon [63] in his review reported the maximum figure of merit reached for both p-type and n-type HH alloys is close to 1.5 until the year 2018. Skutterudites had lower thermal conductivities, whereas the HH alloys had the advantage of high-power factors [64]. Depending on the application requirement appropriate TE material must be selected.

### 3.3. High-Temperature Thermoelectric Materials

High-temperature thermoelectric materials are used in applications at operating temperatures higher than 900 K (silicon, etc.). Several silicon-based alloys (nanocrystalline silicon, magnesium silicide manganese silicide, iron disilicide, cobalt monosilicide, etc.) and few low-toxic Zintl compounds have been developed to reduce the cost and increase its availability. These can be utilized in thermal power plants, industries such as steelworks, glass production, etc., and deep space applications.

#### 3.3.1. Nanocrystalline Silicon-Based TE Materials

Silicon is the second-most abundantly found element on Earth and is non-toxic, making it unproblematic from scarcity, safety, and environmental perspectives. The synthesis, doping, etching, and machining of silicon are well established and it is a simple and the most studied model in material physics. Undoped and single-crystalline silicon have extremely high room temperature thermal conductivity (156 WK^−1^m^−1^) [65]. Although heavy doping has considerably reduced the thermal conductivity (~80 WK^−1^m^−1^ at room temperature), it is still very high to be used as TE material [66]. Nano-structuration of silicon has improved its thermoelectric properties significantly by reducing the lattice thermal conductivities without affecting the electrical conductivities [67]. Narducci et al. reviewed the literature that improved the ZT of Si by increasing the power factor and suggested energy filtering could play a remarkable role in improving the power factor of highly doped nanocrystalline silicon [68]. Thermal conductivities less than 10 WK^−1^m^−1^ and ZT~0.04 at room temperature were observed in the case of nanocrystalline hydrogenated Si thin films prepared by post-deposition thermal annealing [69]. The reduction in thermal conductivities had a better effect on the ZT than the optimization of doping concentrations. Heavily boron-doped nanocrystalline Si films deposited by plasma-enhanced chemical-vapor deposition (PECVD) and hot-wire chemical- vapor deposition (HWCVD) led to the low thermal conductivity of <1 WK^−1^m^−1^ at 300 K. Thus, annealing is the key and better understanding of the grain growth process in nanocrystalline Si with smaller grain sizes and minimized grain growth is required. Schierning [70] conducted a state-of-the-art review on the nanocrystalline Si covering the physical background and different nanostructures such as porous nanomeshes, nanowires, and nanocrystalline bulk and their TE properties. Very high ZT values close to unity (~1) were reported in the case of 20 nm nanowire-silicon materials with p-type doping of 7 × 10^19^ cm^−3^ [71,72]. Shiomi et al. [73] reported on the engineering of phonon transport properties in nanocrystalline Si and suggested a further reduction in thermal conductivities is feasible by controlling the oxygen content during nanostructure formation. Many recent works with the help of theoretical modeling showed the effect of embedded superlattice barriers [74], energy barriers [75], and synergy between charge neutrality, defects, and energy filtering [76] during annealing on improving the thermoelectric properties of the nanocrystalline Si. With further improvements, nanocrystalline Si will prove to be a low-toxic and earth-abundant promising TE material.

**Figure 6:**
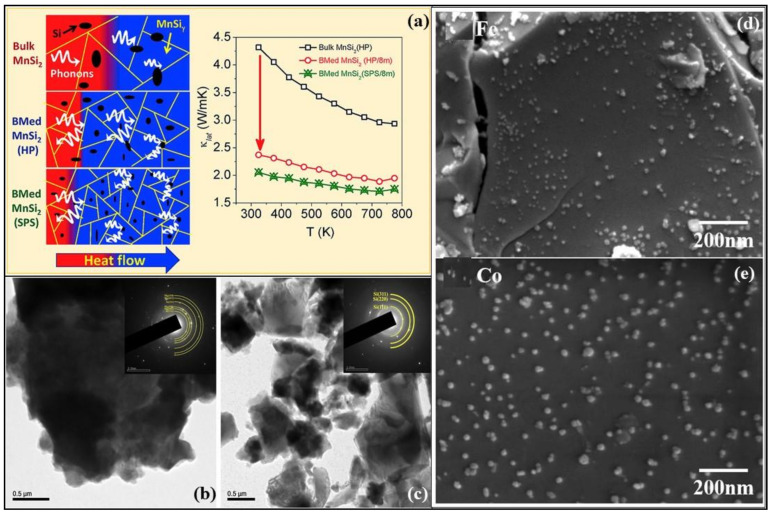
(**a**) Schematic illustration of scattered mid-to-long wavelength phonons by increased grain boundaries of bulk and ball milled (BM) higher manganese silicide embedded with silicon nanoparticles synthesized by hot uniaxial pressing (HP) and spark plasma sintering (SPS). Also, the temperature dependence of lattice thermal conductivities of bulk MnSi_2_ (HP), ball-milled MnSi_2_ (HP), and ball milled MnSi_2_ (SPS) is shown, Reproduced with permission from [77]. Copyright © 2019, Elsevier, (**b**,**c**) TEM images of milled magnesium-silicon mixture and silicon-germanium alloy powder, Reproduced with permission from [78]. Copyright © 2018, Elsevier, (**d**,**e**) SEM images of iron (Fe) and cobalt (Co) nanoparticles in the hybrid powder of MnSi_1.787_Al_0.01_, Reproduced with permission from [79]. Copyright © 2020, Elsevier.

#### 3.3.2. Manganese Silicide Alloys

**Figure 7:**
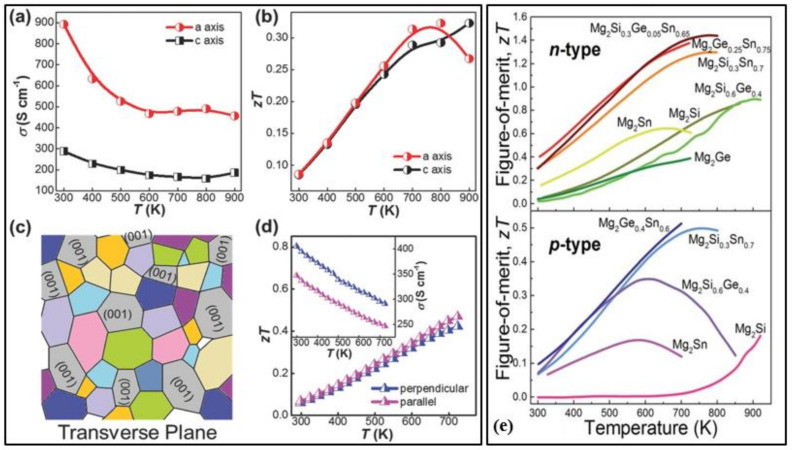
(**a**) Electrical conductivity (σ) of higher manganese silicide (HMS) crystal. (**b**) The estimated dimensionless figure of merit (ZT) of HMS single crystal. (**c**) Schematic diagram of anisotropy of as-sintered HMS pellets in the transverse plane. (**d**) ZT of as-sintered HMS pellet perpendicular and parallel to pressing direction, respectively, inset showing σ along with the two directions, Reproduced with permission from [84]. Copyright © 2018, Wiley Online Library. (**e**) Temperature dependence of figure of merit (ZT) of p-type magnesium-based thermoelectric materials (Mg_2_Ge, Mg_2_Sn, Mg_2_Si, Mg_2_Si_0.6_Ge_0.4_, Mg_2_Si_0.3_Sn_0.7_, Mg_2_Ge_0.25_Sn_0.75_, and Mg_2_Si_0.3_Ge_0.05_Sn_0.65_) and n-type magnesium-based thermoelectric materials(Mg_2_Sn, Mg_2_Si, Mg_2_Si_0.6_Ge_0.4_, Mg_2_Si_0.3_Sn_0.7_, Mg_2_Ge_0.4_Sn_0.6_), Reproduced with permission from [85]. Copyright © 2018, Royal Society of Chemistry.

Manganese and silicon are two prominent Earth-abundant, environmentally-friendly, and cost-effective thermoelectric materials. High purity higher manganese silicide (HMS) materials of the minimal composition of MnSi_1.80_ can be synthesized either by melting or ball mining methods [80]. Figure 6a shows the schematic illustration of scattered mid-to-long wavelength phonons by increased grain boundaries of bulk and ball milled (BM). Higher manganese silicide embedded with silicon nanoparticles synthesized by hot uniaxial pressing (HP) and spark plasma sintering (SPS) [77]. It can also be seen the lattice thermal conductivities have considerably lowered due to the ball milling of the HMS. The various HMS materials have good electrical, mechanical properties and degenerate semiconductor behavior along with relatively low lattice thermal conductivity by the quasi-1D crystal structure [81]. Anisotropic properties have been observed in both polycrystalline and single-crystal HMS. Figure 7a,b,d show the electrical conductivities and figure of merit values of the HMS material. The peak figure of merit ZT of pristine polycrystalline HMS (ZT ≈ 0.4 at 700 K) is larger than that of single-crystal HMS. Re-supersaturation has increased the peak ZT of p-type HMS to ≈1 at 920 K, and W/Fe-supersaturation for n-type HMS has resulted in peak ZT of ≈0.5 at 700 K [82,83]. These two methods were executed by combining the high-temperature melting method with liquid quenching. This process of enhancing ZT and HMS performance has the most promising results for further realizations. Figure 7c presents the schematic diagram of anisotropy of as-sintered HMS pellets in the transverse plane. Point defect scattering via heavy doping by large mass elements such as tungsten and rhenium has reduced the lattice thermal conductivity [84]. The power conversion efficiency (η) of HMS thermoelectric modules has reached more than 7%. Higher efficiencies up to 12% can be gained by stacking this HMS material with Bi-Te based thermoelectric modules [84]. The drawbacks of this proposed method are:

(1) The element rhenium (Re) is a rare-earth material, thereby increasing the cost.
(2) Low chemical stability and can be enhanced by silica-based glass-ceramic coating.
(3) Low ZT value and can be boosted for p-type HMS by employing various strategies of further reducing the thermal conductivity and highlighting the electrical properties.
(4) Power conversion efficiency can be improved by the proper assembling of modules and better design.

#### 3.3.3. Magnesium Silicide (Mg-Si) Alloys

CaMgSi and Mg_2_Si are prominent thermoelectric materials due to their non-toxicity, low material density, natural abundance, and easily modifiable electronic structure. Mg_2_Si has low thermal conductivity and high Seebeck coefficient but lags in its electrical conductivity in the medium to the high-temperature range of 300–700 K. The reason for low electrical conductivity is due to the decreased mobility of charge carriers. This can be negated by appropriate doping and alloying. To find the appropriate alloy compound, VKK Zaitsev conducted series of detailed tests on Mg^2^B^IV^(B^IV^ = Si, Sn, Ge) compounds in the temperature range 300–870 K, and a highly reliable and reproducible ZT value of 1.1 was reported in the case of the n-type solid solution of Mg_2_Si_1−x_Sn_x_ [86]. Mg_2_Sn and Mg_2_Si are two indirect bandgap materials that have their respective low-lying conduction band edges reserved. Effect of band convergence on the thermoelectric properties in a series of Mg_2_Si_1−x_Sn_x_ solutions doped with a negligible amount of antimony showed exceptionally high ZT values of 1.3 near 700 K at x~0.7 [87]. This study suggested that achieving valley degeneracy by varying the compositions is an efficient strategy for better thermoelectric properties. The materials synthesized by high energy mechanical alloying and then sintering of mechanically alloyed powder at 973 K for 20 min resulted in a record-high ZT value of 1.4 for n-type Mg_2_Si_0.4_Sn_0.6_ [88]. The recent progress of both p-type and n-type magnesium silicide alloys is given in Figure 7e.

#### 3.3.4. Chromium Disilicide (CrSi_2_)

The chromium extracted from chromite is available abundantly in Nature, has low-toxicity and good thermal stability at high temperatures, mechanical stability, and is resistant to oxidation in air. Dasgupta et al. [89] synthesized CrSi_2_ using mechanical alloying with a molar ratio of 1:2 for Cr-Si powders using stainless steel media under various milling conditions and reported a maximum ZT~0.2 at 600 K in case of minimum secondary phases. The synthesized material showed good oxidation resistance and thermal stability up to 900 K. The primary issue was the formation of mono silicide of chromium due to iron contamination. Another work emphasized the synthesis of a solid solution of CrSi_2_/5 wt% MnSi_1.73_ by spark plasma sintering to reach a ZT value of ~0.29 at 673 K [90]. The reduction of grain size can be achieved by adding nanometer-sized metallic microstructures. The addition of tungsten disilicate (WSi2) by powder metallurgical process to form composites of Cr-rich hexagonal (Cr, W)Si_2_ phase and the W-rich tetragonal (W, Cr)Si_2_ phase enhanced the phonon scattering, thereby resulting in lower thermal lattice conductivity [91]. The tungsten-doping did not have any effect on the power factor, but a high ZT of 0.3 at 700 K was noted. The optimum nanocomposite composition CrSi2/7.5 wt%SiGe was realized by spark plasma sintering with the dispersion of SiGe nanoparticles(crystalline size ~12 nm) and enhanced ZT of 0.32 at 673 K was registered [92]. The ZT of CrSi_2_ is very low when compared to other silicides but with further improvement in its thermoelectric properties, it can act as a good thermoelectric material.

#### 3.3.5. Other Silicides

A novel geo-abundant and low-toxic thermoelectric material consisting of silicon-boron (Si_96_B_4_) alloy dispersed with SiC nanoparticles are synthesized using spark plasma sintering with peak ZT ~0.27 at 1123 K for Si_96_B_4_/1 wt% SiC nanocomposites [93]. Some other silicides have been proposed over the last two decades, such as iron disilicide (FeSi2), cobalt mono-silicide (CoSi), strontium di-silicide (SrSi2) alloys, etc. that are low-toxic and abundantly available. Yet, the thermoelectric properties such as the figure of merit (ZT), lattice thermal conductivity, and electrical conductivities are not encouraging and less than 0.1(~<0.1).

#### 3.3.6. Chalcogenides (Oxides)

The oxide semiconductors have good thermal and chemical stability in the air at high temperatures. However, the high thermal conductivity and low electrical conductivity have limited the value of the figure of merit in the range of 0.1–0.4 for more than 20 years. Ren et al. [94] reviewed oxide-based thermoelectric materials such as p-type BiCuSeO, Ca_3_Co4O_9_, and NiO, and n-type SrTiO_3_, ZnO, and In_2_O_3_. Table 3 summarizes the recent progress for both p-type and n-type oxides.

Oxides based on Earth-abundant thermoelectric materials are low-cost and easily processable, but they are not suitable candidates as compared with covalent alloys since they have narrowband ionic bonding, low carrier concentration, and mobility. The thermal conductivities must be reduced, and electrical conductivities must be enhanced to attain higher ZT values. Decreasing thermal conductivity is easier than modifying electrical conductivity. This can be done by improving the phonon scattering with the application of nanostructures and doping with heavy elements. The strategies for improving the performance of oxide-based thermoelectric materials are nanostructuring, heavy element doping, and band engineering.

#### 3.3.7. Low Toxic Zintl Compounds

**Figure 8:**
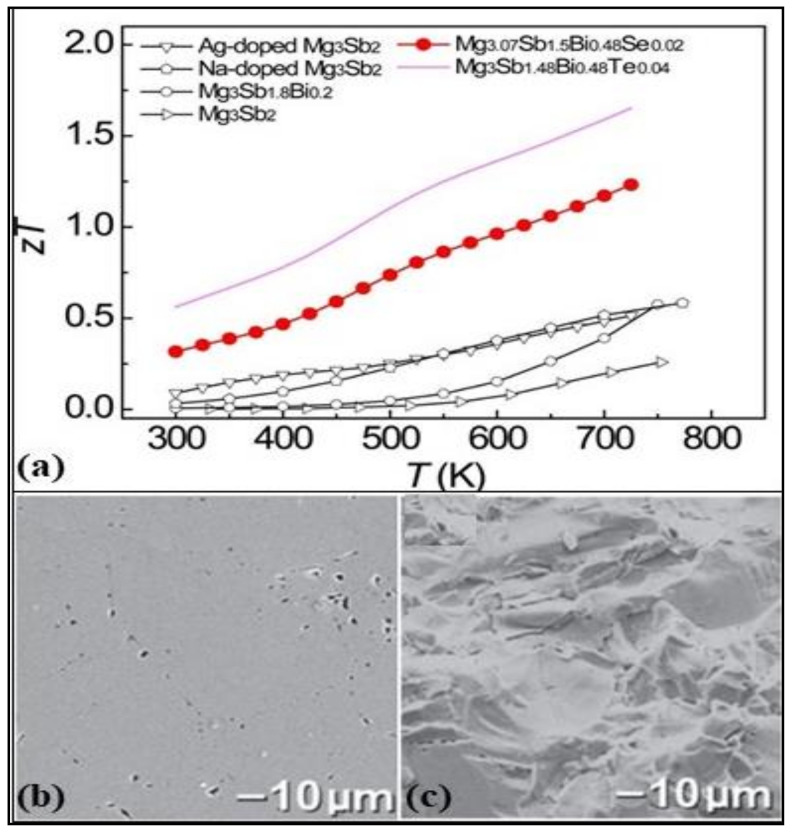
(**a**) Temperature dependence of figure of merit for various Mg_3_Sb_2_-based Zintl compounds: Silver doped Mg_3_Sb_2_, sodium doped Mg_3_Sb_2_, Mg_3.07_Sb_1.5_Bi_0.48_Se_0.02_, Mg_3_Sb_1.48_Bi_0.48_Te_0.04_, Mg_3_Sb_1.8_Bi_0.2_, Mg_3_Sb_2_, Reproduced with permission from [98] Copyright © 2017, ACS Publications. (**b**,**c**) SEM of Ca_4.75_Na_0.25_Al_2_Sb_6_ with 98% theoretical density value (polished surfaces (**b**) and fracture surfaces (**c**)) and grains as large as 50 µm in diameter are revealed, Reproduced with permission from [97] Copyright © 2010, Wiley Online Library.

“Zintl phase” are intermetallic compounds named after the German chemist Eduard Zintl. The main features of these intermetallic phases are their covalent and ionic bonds. The chemical structure of the Zintl phase is A_a_X_x_, where A is active or electropositive metal from groups 1 and 2, and X is noble or electronegative semimetal or metal from groups 13, 14 and 15. From its discovery, beginning from binary to ternary phases, the Zintl phases have expanded to the inclusion of transition and rare earth materials. These materials have potentially useful thermoelectric properties, mainly due to the “electron crystal” electronic structure of the Zintl anions and the “phonon glass” feature of Zintl cations. Recently, few Zintl phases have also been formulated using earth-abundant and low-toxic elements such as Ca_3_AlSb_3_, Mg_3_Sb_2_, CaAl_2_Si_2_, etc. Zevalkink et al. [95] were the first to propose a p-type semiconductor thermoelectric material Ca_3_AlSb_3_ with a ZT of 0.8 and lattice thermal conductivity of 0.6 at 1050 K. The study of two p-type Zintl phases Ca_5_Al_2_Sb_6_ and Ca_3_AlSb_3,_ showed a very low lattice thermal conductivity of 0.5 W/mK and peak ZT of 0.41 at a doping level of 7 × 10^19^ cm^−3^ due to the complex cell structure [96]. These properties can be further enhanced by Na-doping the Ca_5_Al_2_Sb_6_ to formulate a single parabolic band model of composition Ca_4.75_Na_0.25_Al_2_Sb_6_ to reach ZT > 0.6 at 1000 K [97]. Figure 8b,c show the SEM images of the sodium-doped Zintl compound (Ca_5_Al_2_Sb_6_) in case of polished and fracture surfaces with theoretical densities of 98%. Large grains of diameter up to 50 µm can be seen.

Another Zintl compound, Mg_3_Sb_2_, is a low-toxicity and inexpensive thermoelectric material that relies on the “phonon-glass electron crystal” characteristics. Doping the Mg_3_Sb_2_ with 0.2 at.% iso-electronic bismuth ions at the Sb site has enhanced the ZT from 0.26 to 0.6 at 750 K(for composition MgSb_1.3_Bi_0.2_) [99]. The Mg_3_Sb_2_-based Zintl compound doped with 1.25 at.% Na on Mg realized a maximum ZT of 0.6 at 773 K for nanostructured Mg_3-x_Na_x_Sb_2_ at x = 0.0125 [100]. Further doping this Zintl compound with selenium, as well as bismuth, has further increased the ZT value to 1.23 at 725 K for the chemical composition Mg_3.07_Sb_1.5_Bi_0.48_Se_0.02_ [98]. Few Zintl phases, as discussed, are potential candidates for heat recovery applications and have added benefits of low toxicity and inexpensiveness. Further research is required to improve the thermoelectric properties and be achieved by fine-tuning the doping concentration with minimum heavy element doping. The ZT values in the intermediate to high-temperature range of various Zintl compounds can be seen in Figure 8a.

## 4. Outlook

The focus on advanced nanostructures, with an ultimate goal towards the materials made from sustainable, Earth-abundant, economic, and non-toxic resources to replace conventional compounds doped with rare-earth elements, is definitely a good subject for future study. To fully explore this multidisciplinary research, a combined computational-experimental approach is necessary. There is a need to utilize the multidisciplinary (materials science, physics, engineering) collaborative environment to design truly extraordinary TE materials by grain boundary and interface engineering. The general approach should be to independently optimize the doping, electronic band structure, and thermal conductivity of complex TE semiconductor composites to maximize the ZT. The interaction between the material’s parameters such as defects, alloy composition, and microstructure should be understood by modifying the material properties such as electron concentration (doping), effective mass, and lattice thermal conductivity (increasing the ratio µ/κl of quality factor that determines ZT).

The improvement in thermoelectric properties mainly relies on two factors, including minimizing the thermal lattice conductivities and increasing the electrical conductivity. The thermal conductivity can be reduced by (a) nanostructuring and (b) complex crystal structures. In fact, thermal conductivity comprises two parts: *κ_e_* (due to heat-carrying electrons or holes charge carriers) and *κ_L_* (lattice thermal conductivity). 2D structures such as superlattices and quantum wells and 1D structures like nanowires have been shown to significantly enhance the ZT by lowering the lattice thermal conductivity. Even though the complex crystal structures have limited phonon mean free path, however, the elucidation and identification of the structural component influencing the transport properties are tricky.

To increase the electrical conductivity in the higher energy levels, resonant doping has proven an effective technique; however, successful identification of optimal resonant dopants is a herculean task. The high Seebeck coefficient is originated from the heavier density-of-states effective mass. This, in turn, leads to an enrichment of the thermoelectric properties of the material by the rise in the Seebeck coefficient and power factor. Zero-dimensional Point defects, one-dimensional linear defects, two-dimensional planar defects, and three-dimensional bulk defects are also induced in the materials for optimizing their thermoelectric properties. The introduction of these defects influences the physical parameters of the material and tunes the carrier concentration causing an enhancement in both the Seebeck coefficient and electrical conductivity. Research on complex point defects impacting the band structure is necessary for further expansion of thermoelectric applications.

CuI is considered a potential candidate for low-toxic and Earth-abundant materials. Nevertheless, as far as the CuI is concerned, this transparent TE material demonstrated low electrical conductivities resulting in inferior thermoelectric properties. Also, the CuI thin films were found to be electrically unstable at beyond 80 °C temperatures. Among the low-toxic materials, the copper sulfides (with a mean ZT value >1.2) exhibited great potential for the intermediate and high-temperature range. However, the major concern is the volatilization of sulfur that occurs during synthesis, stacking, and higher temperatures, thereby affecting the material stability. The loss of sulfur leads to changes in the nominal stoichiometries making reproducibility challenging to achieve. The mobile copper ions migrate and form a layer at one end of the thermoelement under the electric field created when the device is operated at a temperature gradient resulting in lower performance, cracking, and compositional changes. Further study is required in search of other compatible materials for module construction with better chemical stability and mechanical properties per practical device applications.

Silicon-based TE materials are a perfect alternative to high-toxicity and rare TE materials in the high-temperature range applications. These materials are low toxicity, low-cost, easy to fabricate and recycle and have better physical, chemical, thermal, and mechanical stability. Current research shows that these materials have a mean ZT > 1.2. Up to date, all the research towards silicon-based TE materials is based on lowering the lattice thermal conductivities for improving the ZT values. The focus now needs to be shifted on enhancing the thermoelectric power factor by enhancing the Seebeck coefficient. Quantum confinement engineering, distortion of electronic band structure by resonant impurity states, hot carrier filtering, or improving the number of carrier pockets near the band edges (larger band multiplicity) are some suggestions along this direction.

Nevertheless, to date, there are no vivid roadmaps for accomplishing many of these goals. Computational advancements and theoretical tools can aid in the quest to discover or innovate new TE materials. Better identification of the underlying factors for efficient correlation of the phonon and electron interactions can also contribute to elevated thermoelectric power. Few TE materials have been engineered using these techniques, and many more are yet to be explored both in theoretical and practical terms. The future high-performance TE materials engineered must have a perfect balance between superior power factors and reduced thermal conductivities.

## Figures and Tables

**Table 1:** Prominent nanostructured copper-iodide (CuI) alloys/composites thermoelectric materials.

Sr.no	Material	Operating Temperature (K)	Synthesis Technique	σ * (Scm^−1^)	K * (Wm^−1^K^−1^)	Seebeck Coefficient (µVK^−1^)	PF * (S^2^σ/µWm^−1^K^−2^)	ZT *_max_	Year/ Reference
1	p-type CuI	300	Sputtering	N/A	0.55	237	375	0.21	2017 [9]
2	p-type CuI	353	Simple Synthesis Vacuum Annealing	N/A	N/A	431	160	N/A	2018 [10]
3	p-type CuI n-type GZO	300	Sputtering Thermal evaporation Solid deposition Vapor method	110	N/A	206	470	0.22	2018 [11]
4	p-type CuI n-type GZO	373	Resistive thermal evaporation	110 142	0.48 2.17	206.0 −60	470 500	0.29 0.07	2019 [12]
5	CuI:Tb (0.05 mol%) NP *	430	Hydraulic pressing Vacuum annealing	~500	~0.5	~700	350	0.28	2020 [13]

* Nomenclature: NP: nanoparticles; NS: nanosheets; σ: electrical conductivity; k: thermal conductivity; PF: power factor; ZT: the figure of merit.

**Table 3:** The maximum figure of merit ZT for various oxide-based thermoelectric materials [94].

	Type of Thermoelectric Material	Max ZT
**n-type**	ZnO-based	0.54 at 1000 K
	SrTiO_3_-based	0.37 at 973 K
	In_2_O_3_-based	0.7 at 1073 K
**p-type**	Ca_3_Co_4_O_9_-based	0.4 at 975 K
	BiCuSeO-based	1.4 at 923 K
	NiO-based	0.075 at 1000 K

## Data Availability

No data available.
